# Does VEGF-targeted active immunotherapy induce complete abrogation of platelet VEGF levels?

**DOI:** 10.1186/s13104-019-4368-z

**Published:** 2019-06-10

**Authors:** Javier Sánchez Ramírez, Mónica Bequet-Romero, Yanelys Morera Díaz, Francisco Hernández-Bernal, Marta Ayala Avila

**Affiliations:** 1 Department of Pharmaceuticals, Center for Genetic Engineering and Biotechnology, Ave 31 e/158 y 190, Playa, P.O. Box 6162, Havana, Cuba; 2 Department of Clinical Research, Center for Genetic Engineering and Biotechnology, Ave 31 e/158 y 190, Playa, P.O. Box 6162, Havana, Cuba

**Keywords:** Specific active immunotherapy, VEGF vaccine, CIGB-247, Platelets, Phase I clinical trial

## Abstract

**Objectives:**

Vascular endothelial growth factor (VEGF) is involved in physiological angiogenesis, but also is considered one of the key factors that promotes tumor angiogenesis. CIGB-247 is a VEGF-based vaccine that has been evaluated in phase I clinical trial patients with advanced solid tumors. This specific active immunotherapy is able to reduce platelet VEGF levels; however it is unknown whether this effect leads to a decrease in VEGF below the levels that can be observed in healthy individuals. The objective of the present study is to investigate platelet VEGF levels in cancer patients vaccinated with CIGB-247, and then compare these values with those obtained in healthy individuals. To achieve this, platelet VEGF levels of 62 cancer patients and 93 healthy individuals were compared. Cancer patients were those individuals recruited in CENTAURO and CENTAURO-2 clinical trials.

**Results:**

Before vaccination, platelets of cancer patients carried more VEGF than the levels seen in platelet of healthy individuals. However, after vaccination, cancer patients had platelet VEGF values within the range established by healthy individuals, indicating that the antibody response elicited by CIGB-247 is not able to induce a complete suppression of VEGF. Vaccination with CIGB-247 helps to normalize VEGF levels within platelets.

**Electronic supplementary material:**

The online version of this article (10.1186/s13104-019-4368-z) contains supplementary material, which is available to authorized users.

## Introduction

Vascular endothelial growth factor (VEGF) is crucial during physiological and pathological angiogenesis. Physiological angiogenesis mediates different processes such as female sexual cycle, wound healing, bone repair and tissue differentiation. Pathological angiogenesis involves tumor angiogenesis, characterized by an increase in VEGF secretion from tumor cells and the number of blood vessels around the tumor. The increment of blood vessels allows an increment of oxygen and nutrients necessary to support tumor growth and its metastasis [1]. For that reason, VEGF has become an attractive target for cancer immunotherapy.

CIGB-247 is a cancer therapeutic vaccine that uses as antigen a recombinant mutated version of human VEGF. This VEGF-based vaccine has two different formulations with the adjuvants VSSP or aluminum phosphate, in both cases with clinical evaluations in cancer patients [2, 3]. Both vaccine formulations showed an excellent safety profile and elicited specific IgG antibodies able to decrease VEGF levels within platelets [2, 3]. “Platelet VEGF” has been chosen as methodology to evaluate the effect of this active immunotherapy on VEGF levels because it was associated with those groups of individuals that exhibited the best specific humoral response, and the variation of “platelet VEGF” showed the strongest negative correlation with VEGF-specific IgG antibody levels [4]. This methodology, known as “platelet VEGF”, is based on the estimation of VEGF within platelets by subtracting the plasma VEGF level from the serum level and dividing this by the platelet count, and then this latter expression is additionally corrected by the hematocrit [5].

VEGF reduction induced by Bevacizumab, a monoclonal antibody directed to human VEGF and approved for the treatment of some tumors [6–10], has been associated with the adverse effects observed for this type of immunotherapy, also denoted as anti-VEGF class toxicities. These adverse effects including hypertension, hemorrhage, gastrointestinal perforation, cardiac events, thromboembolism and proteinuria are considered downstream consequences of inhibition of VEGF-signaling pathways, important in the regulation and maintenance of the microvasculature [1]. The large magnitude of this inhibition is due to the high amount of monoclonal antibody that is administered to achieve a therapeutic effect leading in turn to a high rate of free VEGF abrogation. For example, Karp and colleagues have demonstrated that after 2 h of the Bevacizumab infusion, patients could experience a complete neutralization of their free circulating VEGF [11].

With the aim to investigate the magnitude of platelet VEGF reduction induced by the antibody response elicited in cancer patients vaccinated with CIGB-247, these values were compared with those obtained in healthy individuals. Cancer patients were those subjects recruited in CENTAURO and CENTAURO-2 clinical trials [2, 3], and healthy individuals were carefully selected for this purpose.

## Main text

### Methods

#### Healthy individuals

Healthy individuals comprised 36 women and 57 men. Age ranged between 16 and 72 years (Additional file 1). Individuals with history of cancer, inflammatory diseases, diabetes, sicklemia, anti-inflammatory drug use, or women in menstrual period were excluded from the study. Informed consent was obtained for all individuals in accordance to institutional practice.

#### Cancer patients

This study analyzed cancer patients enrolled in phase I clinical trials: 24 patients from CENTAURO clinical trial (trial registration number: RPCEC00000102) and 38 patients from CENTAURO-2 clinical trial (trial registration number: RPCEC00000155) [2, 3]. Cancer patients were immunized with different antigen doses in combination with adjuvants VSSP or aluminum phosphate, and comprised 41 women and 21 men (Additional file 2). Both clinical trials recruited patients with advanced solid tumors and with different types of malignancies at original diagnosis. Written informed consent was obtained for all patients. Both clinical studies were conducted in accordance with the ethical guidelines of the Declaration of Helsinki and were approved by the hospitals institutional review boards and ethics committees (CIMEQ, Celestino Hernández Robau and José Ramón López Tabranes hospitals) and by the Cuban Regulatory Authority.

#### Human blood samples

Blood samples were taken into an ethylenediaminetetraacetic acid (EDTA) tube or into a serum separator tube containing a serum clot activator. EDTA tube was immediately centrifuged, and serum separator tube was incubated for 1–2 h at 25 ± 3 °C before centrifugation. Tubes were centrifuged for 10 min at 1800*g* and 25 °C [4].

Serum and plasma samples from healthy individuals were stored at − 70 °C and were analyzed between 1 and 22 days after. In case of samples from cancer patients, this time ranged between 4 and 13 months.

Samples from cancer patients were obtained before the initial vaccination (week 0) and after the induction phase (week 13).

#### Platelet VEGF

VEGF concentrations were measured as previously described [4]. All measurements were made by the same analyst and at the same laboratory. Platelet VEGF was expressed in picograms of VEGF/10^6^ platelet and was calculated using the following formula [5]:

1 $${\text{platelet}}\;\;{\text{VEGF}} = \frac{{({\text{serum}}\;\;{\text{VEGF}} - {\text{plasma}}\;\;{\text{VEGF}}) \times (1 - {\text{hematocrit}})}}{{{\text{platelet}}\;\;{\text{count}}}}$$

Platelet counts were performed using an automated hematology analyzer. The variation (Δ) of platelet VEGF levels was calculated using the following formula [4]:

2 $$\Delta = \left[ {\left( {\frac{{{\text{levels }}\;\;{\text{at}}\;\;{\text{week}}\;13}}{{{\text{levels}}\;\;{\text{at}}\;\;{\text{week}}\;0}} } \right) \times 100} \right] - 100\%$$

Based on criteria established by other authors [12], Δ ≤ − 30% was considered a decrease; Δ ≥ 30% was considered an increase; − 30% < Δ < 30% indicated a stability.

#### Statistical analysis

Two-group comparisons of unpaired data were made using the Mann–Whitney test. Wilcoxon-matched pairs test was used to evaluate differences of paired observations. Statistical significance was considered as p < 0.05.

### Results

**Fig. 1:**
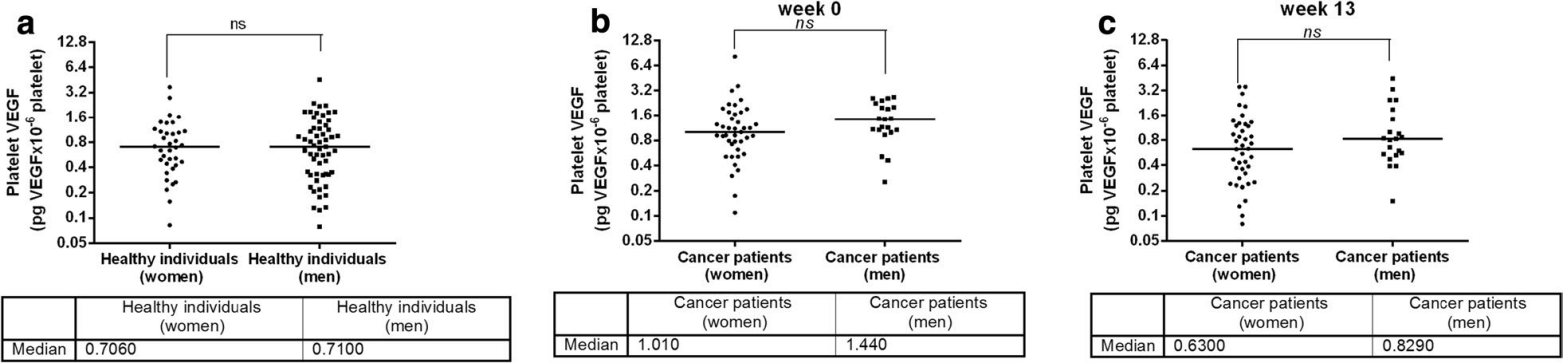
Platelet VEGF levels in healthy individuals and cancer patients analyzed by gender distribution. **a** Platelet VEGF levels in healthy women and healthy men. **b** Platelet VEGF levels before vaccination (week 0) in cancer patients recruited for CENTAURO and CENTAURO-2 clinical trials. **c** Platelet VEGF levels after vaccination (week 13) in cancer patients recruited for CENTAURO and CENTAURO-2 clinical trials. Horizontal bars represent the median values of platelet VEGF. *ns* non-significant

Gender distribution was different between the groups of healthy individuals and cancer patients. To address whether platelet VEGF levels are different between women and men, statistical analysis was performed by gender in both types of subjects: healthy individuals and cancer patients. Platelet VEGF in 36 healthy women moved between 0.082 and 3.719 pg of VEGF/10^6^ platelets with a median value of 0.7060, while platelet VEGF in 57 healthy men ranged between 0.079 and 4.552 pg of VEGF/10^6^ platelets with a median value of 0.7100. Differences between healthy women and healthy men were not found (Mann–Whitney test, *p *= 0.9874) (Fig. 1a). For cancer patients before vaccination (week 0), platelet VEGF in 41 women moved between 0.110 and 8.190 pg of VEGF/10^6^ platelets with a median value of 1.010, while platelet VEGF in 21 men ranged between 0.252 and 2.650 pg of VEGF/10^6^ platelets with a median value of 1.440. Before vaccination, differences in cancer patients between women and men were not found (Mann–Whitney test, *p *= 0.0872) (Fig. 1b). Similar results were observed in vaccinated cancer patients (week 13), where no differences were observed between both sexes (Mann–Whitney test, *p *= 0.1621) (Fig. 1c). All these results indicate that sex is not a parameter with influence on platelet VEGF levels, and it might be possible to make comparisons between groups of individuals with a different gender distribution.

**Fig. 2:**
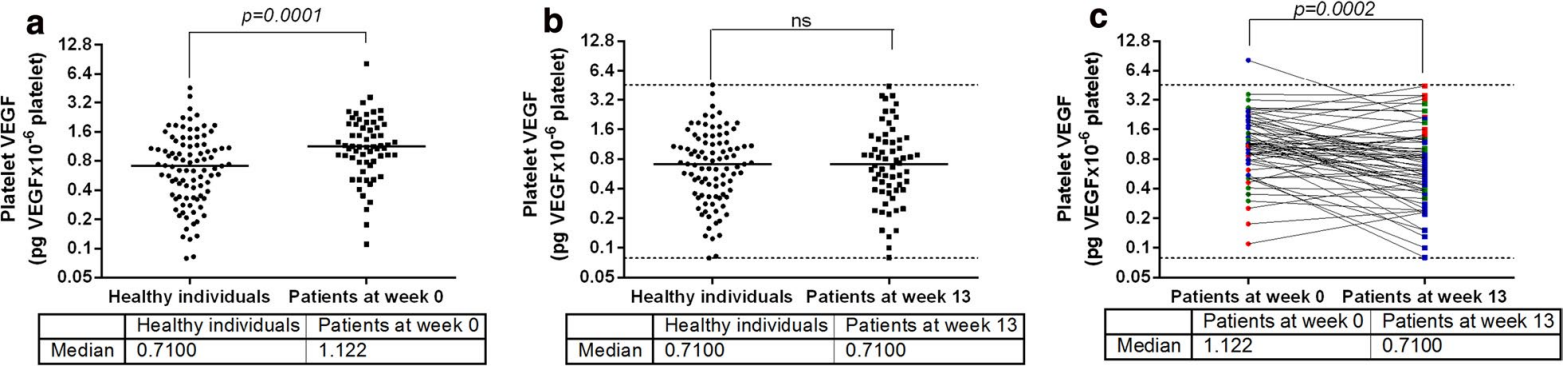
Platelet VEGF levels in healthy individuals and cancer patients. Both groups of subjects included women and men, and patients were those individuals recruited for CENTAURO and CENTAURO-2 clinical trials. **a** Platelet VEGF levels in healthy individuals and platelet VEGF levels in cancer patients before vaccination (week 0). **b** Platelet VEGF levels in healthy individuals and platelet VEGF levels after vaccination (week 13). Horizontal bars represent the median values of platelet VEGF. **c** Platelet VEGF levels in cancer patients before and after vaccination. The variation (Δ) of platelet VEGF levels, expressed in percentages, was calculated using Eq. 2 as described in “Methods”. Blue symbols represent patients with VEGF reduction (Δ ≤ − 30%), green symbols represent patients with VEGF stability (− 30% < Δ < 30%), and red symbols represent patients with VEGF increase (Δ ≥ 30%). Discontinued lines represent the range (maximum and minimum) of platelet VEGF obtained from healthy individuals. Statistical significance was considered as p < 0.05. *ns* non-significant

When healthy women and healthy men were pooled (93 healthy individuals), platelet VEGF ranged between 0.079 and 4.552 pg of VEGF/10^6^ platelets with a median value of 0.7100, while platelet VEGF in 62 cancer patients (women + men) at week 0 ranged between 0.110 and 8.190 pg of VEGF/10^6^ platelets with a median value of 1.122 (Fig. 2a). Before vaccination, platelet VEGF levels in cancer patients were significantly higher than the levels seen in healthy individuals (Mann–Whitney test, *p *= 0.0001) (Fig. 2a), indicating that platelets of cancer patients carry more VEGF than platelets of healthy individuals. However, when these patients were submitted to vaccination (week 13), there were no differences in platelet VEGF levels between vaccinated patients and healthy individuals (Mann–Whitney test, *p *= 0.8351) (Fig. 2b), indicating that after vaccination platelets of cancer patients and platelets of healthy individuals transport similar amounts of VEGF. Platelet VEGF at week 13 moved between 0.080 and 4.418 pg of VEGF/10^6^ platelets with a median value of 0.7100 (Fig. 2b).

Cancer patients included subjects with different primary tumors and treated with different vaccine combinations. Despite these differences, platelet VEGF levels at week 13 were significantly lower than the levels detected at week 0 (Wilcoxon-matched pairs test, *p *= 0.0002) (Fig. 2c). Vaccination was effective in 32 patients (Δ ≤ − 30%, blue symbols) while 18 patients did not experience any changes on platelet VEGF levels between weeks 0 and 13 (− 30% < Δ < 30%, green symbols). The remaining 12 patients increased their platelet VEGF levels (Δ ≥ 30%, red symbols). As shown Fig. 2b, c, the values of platelet VEGF of vaccinated cancer patients fell within the range observed in healthy controls.

### Discussion

VEGF is carried by different blood cell components, but platelets are considered its major physiological transporter [13]. There are several approaches to estimate indirectly the VEGF content inside platelet, and they are described by different methodologies, most of the cases grouped under the same term known as “platelet VEGF”. For example, “platelet VEGF” can be determined as serum VEGF normalized by the patient’s platelet count [14] or by subtracting the plasma VEGF level from de serum level and dividing this by the platelet count [15] or this latter expression additionally corrected by the hematocrit [5]. Despite the term “Platelet-VEGF” is not uniformly used in the literature, these measurements have become an important tool in clinical monitoring of patients submitted to anticancer therapies [14, 16].

Antiangiogenic agents like Bevacizumab have shown promising clinical results, although some adverse effects have been associated to the treatment. Adverse effects observed with the use of Bevacizumab, a monoclonal antibody that shows a high affinity for human VEGF [17], have indicated that VEGF has an important role in normal physiology [18]. Bevacizumab administration induces a drastic and significant decline in VEGF bioavailability [11] with the aim to negatively impact on tumor burden or tumor angiogenesis. Specific active immunotherapy is another alternative to reduce VEGF levels, and CIGB-247 is a VEGF vaccine included within this type of strategy.

Using this VEGF vaccine, a significant decrease on platelet VEGF levels has been observed in the groups of patients vaccinated with 400 or 800 µg of antigen in combination with VSSP as adjuvant, or 400 µg of antigen mixed with aluminum phosphate [2, 3]. However, the specific antibody response elicited by CIGB-247 is not able to induce a complete suppression of VEGF; some VEGF levels remain circulating within platelets to maintain normal adult vasculature or other VEGF-dependent normal physiological processes. Complete abrogation of VEGF is not possible to achieve because the polyclonal antibodies elicited by CIGB-247 is a regulated response that is gradually generated over time. During the course of this response, the degree of VEGF-blocking activity increases as the levels of antibodies is increased. This blocking activity on VEGF levels happen at normal physiological concentrations of polyclonal antibodies. These levels of active polyclonal antibodies are much lower than the levels of Bevacizumab administered during intravenous infusion. This fact could explain why the anti-VEGF class toxicities have not been reported in CENTAURO and CENTAURO-2 clinical trials and why this active immunotherapy has an excellent safety profile [2, 3].

The clinical success of anti-angiogenic agents like Bevacizumab is undeniable, and could be considered a strong “attack” therapy. In some cases, this passive immunotherapy is interrupted due to the severity of adverse effects, opening a new window to treat these patients with CIGB-247, an active immunotherapy that has a very safety profile [2, 3]. Additionally, the treatment with CIGB-247 could be beneficial for patients due to generation of VEGF-specific cytotoxic CD8+ cells [2] with potential ability to eliminate cancer cells. CIGB-247 is being evaluated in phase II clinical trials in patients with hepatocellular carcinoma or ovarian cancer (RPCEC00000237 and RPCEC00000246 respectively). The reference values established from healthy individuals and presented here could be very useful to continue studying in a larger group of patients with the same type of tumor whether the vaccine decreases platelet VEGF levels below these limits.

### Conclusions

This study has demonstrated that after using CIGB-247, the magnitude of platelet VEGF reduction can be considered as an incomplete blocking effect. Platelet VEGF levels of vaccinated cancer patients can be detected within the physiological range and not below this limit. This investigation also found that vaccination contributes to normalize VEGF levels within platelets.

## Limitations

This study has some limitations; at first, the physiological range was established using a relatively low number of healthy individuals. Second, the number of cancer patients is limited in size, as well as the aspect that patients had different types of malignancies at original diagnosis.

## Additional files

**Additional file 1.** Measurements in healthy individuals.

**Additional file 2.** Cancer patients data.

## Data Availability

Some data analysed during this study can be found here: 10.1016/j.heliyon.2018.e00906. The datasets generated during the current investigation are available in this published article and its additional files.

## Abbreviations

VEGF: vascular endothelial growth factor
CIGB-247: VEGF-based vaccine
EDTA: ethylenediaminetetraacetic acid
